# Complication of nasal piercing by *Staphylococcus aureus endocarditis*: a case report and a review of literature

**DOI:** 10.1186/1757-1626-3-37

**Published:** 2010-01-25

**Authors:** Battagin Giuliana, Sarmati Loredana, Sordillo Pasquale, Picchi Giovanna, Calisti Giorgio, Ceccarelli Laura, Antonio Pellegrino, Nardi Paolo, Chiariello Luigi, Andreoni Massimo

**Affiliations:** 1 Department of Clinical Infectious Diseases and Cardiac Surgery, Tor Vergata University Hospital, V. Montpellier 1, 00133, Rome, Italy

## Abstract

Body piercing, a growing trend especially in young people, is often complicated by severe infections. We present a case of acute bacterial endocarditis by *Staphylococcus aureus *complicated by multiple cerebral, kidney, spleen embolisms in a young girl, with no known previous cardiac abnormalities, following the piercing of nasal septum. This case highlights the importance of education of patients with and without structural heart disease to the potential dangerous and even life threatening infectious complications of piercing, and stimulate further discussion on the possibility of antibiotic prophylaxis of such procedures.

## Introduction

Body piercing is a type of self-expression that has been both greatly accepted and widely performed in modern society. Even in healthy individuals, significant health risks are associated with this type of physical adornment, including infection, pain, bleeding, hematoma and cyst formation, allergic reactions, hypertrophic scarring, and keloid formation. Infection severity ranges from local infections (e.g., impetigo and cellulitis) to more extensive or even systemic infections such as osteomyelitis, toxic shock syndrome, and bacteremia. Numerous surveys carried out in different countries have demonstrated a high prevalence of body piercing and a related risk of subsequent, sometimes even severe, complications [1-4].

Although there is no denominator with which to assess the real risk, many papers provide evidence of a strict pathogenetic correlation between piercing and the development of endocarditis, which should stimulate further discussion regarding the suggested antibiotic prophylaxis before piercing practice [5,6].

This case report highlights the importance of educating both the general population and patients with structural heart diseases about the potential risks associated with body piercing procedures.

## Case presentation

An 18-year-old Caucasian, Italian girl, with a history of occasional use of inhaled drugs, was admitted to the Tor Vergata University Hospital emergency room because of fever, nausea, malaise, and difficult walking. The patient had been healthy until three days before admission, when she developed fever. She had no history of intravenous drug use, recent dental procedures, heart murmurs or cardiac abnormalities. Six months earlier, she had had her nasal septum pierced, and subsequently she frequently removed her jewellery to hide it from her parents, putting it back in place again later.

On admission, physical examination revealed hypotension, dehydration and a clinical presentation suspicious for sepsis, with multiple Janeway lesions on the volar surface of her feet and hands, furunculosis on the legs and Osler lesions on the periungual area of two fingers. Neurological examination revealed a mild cognitive deficit, left extremity weakness and incapacity to coordinate the left side of her body. At the level of the nasal septum, an erythematous, ulcerative bleeding lesion was present in the mucosa previously perforated by a nasal piercing.

**Figure 1:**
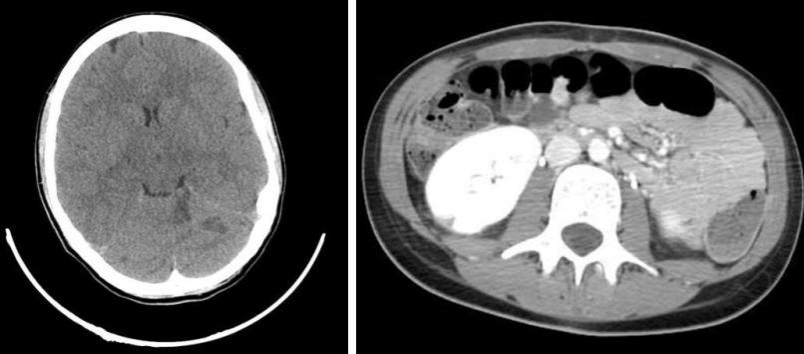
**(a) Evidence of three contrast-enhancing lesions within the left cerebellar side on a CT central nervous system scan**. **(b) **right kidney infarction.

**Figure 2:**
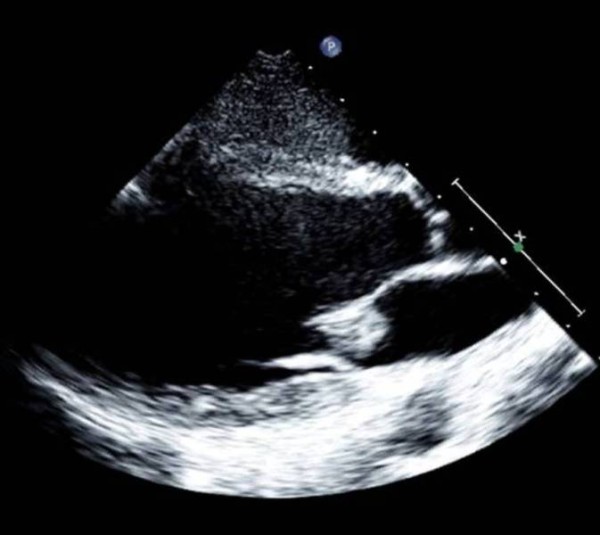
**Transthoracic echocardiogram showing a mitral vegetation of 15 × 13 mm on the anterior cusp**.

Blood cultures obtained on admission were strongly positive for a blood-borne staphylococcus aureus (*S. aureus*) infection. Whole body computed tomography (CT) (Figure 1a) revealed two contrast-enhancing lesions within the left cerebellar and occipital cerebral regions, suggestive of septic emboli. Multiple similar lesions were observed in the liver, spleen and kidneys (Figure 1b). Fundus oculi revealed septic emboli (Roth lesions). Serial trans-thoracic echocardiography showed a pedunculated 15 × 13 mm vegetation sitting on the anterior mitral leaflet at the level of the postero-medial commissure, which was considered embolizing, according to the brain magnetic resonance imaging (MRI) findings. A mild mitral valve incompetence was also detected (Figure 2).

Broad-spectrum antibiotic therapy with gentamycin, vancomycin and linezolid was initiated. When the antibiotic assay demonstrated a methicillin-sensitive *S. aureus *strain (MSSA), vancomycin was replaced with oxacillin. After 6 days of treatment, the patient remained febrile, with blood cultures still positive for MSSA, prompting the addition of tigecycline to the antibiotic regimen.

**Figure 3:**
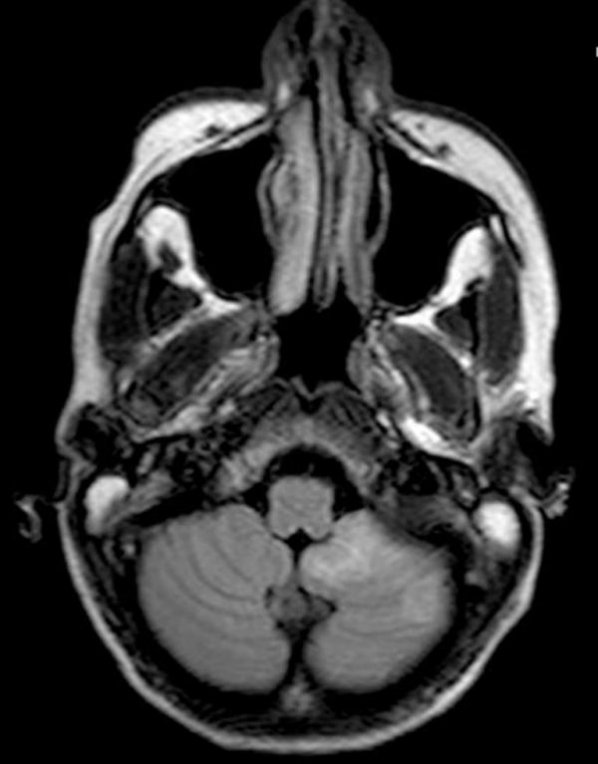
**Brain magnetic resonance imaging (MRI) revealing left side cerebellar abscesses**.

On the eighth day following the admission, a whole body CT demonstrated reduction in spleen, kidney and liver size and number of lesions. However, MRI of the brain revealed abscessualization of the cerebellar lesions (Figure 3).

On day 11 of treatment, the patient's condition improved and fever had disappeared. In order to remove the vegetation dangerously fluctuating in the left atrial cavity and correct the mitral valve incompetence, although the duration of antibiotic therapy was deemed not yet complete, surgical intervention was performed on April 1, 2008 by means of median sternotomy, cardiopulmonary bypass and antegrade tepid blood cardioplegia. The vegetation was removed and a posteromedial commissuroplasty was performed.

The immediate postoperative course was uneventful, and echocardiographic controls revealed a well functioning mitral valve. However two weeks after surgery heart failure developed and the patient presented with increasing dyspnoea, tachycardia and a new systolic murmur. Transthoracic echocardiography revealed massive mitral insufficiency with concomitant pulmonary hypertension. For these reasons, a new surgical procedure was required and performed on April 23, 2008. The anterior leaflet of the mitral valve, partially destroyed by the infection, was repaired with an autologous pericardial patch and an A3-P3 edge-to-edge valvuloplasty technique was employed to complete the mitral valve repair. A prosthetic ring was then sutured to the posterior segment of the mitral annulus to stabilize the repair.

Postoperatively, linezolid, tigecycline and oxacillin were administered for 40 days. Subsequently ceftriaxone and rifampicin were administered until the cerebellar lesions were no longer evident on MRI.

The patient was uneventfully discharged on the fortieth postoperative day. A long term antibiotic treatment was prescribed in order to completely eradicate the cerebellar abscesses. Serial echocardiographic examinations were performed. At one year follow-up the patient appeared to have fully recovered and the mitral valve was functioning showing only trivial regurgitation.

## Conclusion

In our patient staphylococcal endocarditis most likely subsequent to a nasal piercing occurred. Although no bacterial culture was obtained from the nasal septum lesion, in absence of any other evident source of bacteraemia, one can suppose that endocarditis was triggered by organisms that colonized around or adhered to the jewellery, favored by the repeated trauma occurring when jewellery was periodically removed. Nasal cavities are naturally bacteria-laden and anterior nares are the most frequent site of human colonization by *S. aureus*. Our patient presented with an inflamed, ulcerated lesion at the nasal septum, probably subsequent to the continual removal of her piercing device. This maneuver, and the associated occasional intranasal drugs use, could have favored staphylococcal bacteremia with subsequent development of severe acute endocarditis.

Examples of the increasing prevalence of body piercing are ubiquitous. A survey on piercing at sites other than the earlobes in the English population showed a 10% prevalence of body piercing in adults (16 years and over), with complications observed in 31% of cases, and with 0.9% of such complications requiring hospital admission [4]. Other surveys performed in Australia, Germany, and the United States have reported similar prevalence [1-3]. Despite increasing diffusion of this type of body art, a very limited number of countries regulate non-traditional body piercing. A European law (UNI EU 18.10 September 18, 2002) regulates the practice of piercing, however it is not restrictive: some individuals learn the craft through apprenticeship-type arrangements, but most individuals performing body piercing are self-trained. In addition, no standard of informed consent for piercing recipients currently exists. There is no precise regulation, despite the significant risks that can be associated with this practice. The U.S. National Institutes of Health have identified piercing as a possible vector for blood-borne hepatitis transmission. Many physicians have also suggested that piercing may be a risk factor for the transmission of human immunodeficiency virus, although, to date, no specific case reports of such transmission have been described. Increased risk of tetanus, tuberculosis [7-9] and other infectious diseases have been demonstrated to be associated with piercing. Furthermore, numerous piercing sites have been associated with systemic infectious complications. At present, numerous cases of endocarditis have been reported in the medical literature [10-20], indicating that the number of systemic severe infections referred to piercing is increasing with time. The most common aetiological agents in the described cases of endocarditis have been staphylococci and streptococci, but *Haemophilus aphrophilus *and *H. parainfluenza *have also been reported as causative agents of systemic infection. In all cases, the aetiological agent was obtained from blood cultures, but only in four a bacterial culture was found positive at the piercing site. In five cases, the patient had a previously known heart valve disease. In half of cases, symptoms started two to seven days after the piercing had been performed, in the other half several months later.

The present case represents a severe complication of body piercing in a patient with no known previous cardiac abnormalities. Potential infectious complications of piercing may be dangerous and even life threatening and should therefore be well known by users; individuals at high risk for such adverse outcomes should be identified for adequate prevention. In particular, patients with known cardiac abnormalities should be strongly advised against piercing and informed about the danger related to it.

## Consent

The patient and the ethical committee of the hospital gave their approval for the anonymous publication of the clinical case.

## Competing interests

The authors declare that they have no competing interests.

## Authors' contributions

BG, participated in clinical care of patient and drafted the manuscript, SL, infectious diseases specialist, draft and reviewed the manuscript, SP, infectious diseases specialist, treated the patient, PG, participated in clinical care of patient, CG, participated in clinical care of patient, CL, participated in clinical care of patient, PA, cardiac surgery specialist, did cardiac surgery procedures, NP cardiac surgery specialist, did cardiac surgery procedures, CL cardiac surgery specialist, did cardiac surgery procedures, reviewed the manuscript, AM, infectious diseases specialist, reviewed the manuscript, All authors read and approved the final manuscript.
